# Lessons from a Special Service for Public Health, Brazil

**DOI:** 10.3201/eid1510.090654

**Published:** 2009-10

**Authors:** Amelia L. Mayberry, Timothy D. Baker

**Affiliations:** Johns Hopkins University Bloomberg School of Public Health, Baltimore, Maryland, USA

**Keywords:** Brazil, Amazon region, communicable diseases, national health programs, enteric infections, bacteria, parasites, letter

**To the Editor**: Many thanks for your interesting and informative special section on infectious diseases in the Amazon Region (*1*). Your readers should also be interested in a little known, but extremely successful, sustainable health program that had its start in the Amazon.

In 1942, the governments of Brazil and the United States agreed to establish a special service for public health (called the Serviço Especial de Saúde Pública). The purpose of this program was to improve health conditions in key areas in the Amazon, expedite the collection and export of native rubber, and counteract the growing influence of Nazi Germany in Latin America (*2*). The program spread to the Vale do Rio Doce, where there were resources of iron ore, mica, and optical quartz, which were important for the war effort. Although the program eventually moved to all states of Brazil, the Amazon program remained an important activity for ≈50 years before it was integrated into the Brazilian Ministry of Health (*3*).

The program in the Amazon focused primarily on infectious disease. It comprised programs of immunization, provision of small sustainable water systems, development of privy programs (sewer systems in the larger centers of population), malaria control, improvement of residences and living conditions for Chagas disease control, epidemiologic intelligence, and extensive training for auxiliary and professional personnel.

The effects of this program are shown by the increase in life expectancy for all age groups, with an increase of >10 years for those childhood age groups for whom infectious disease control would have the greatest effect from 1939–1941 to 1950–1951 (*4*). This program contains many lessons for the planners of health and disease control projects in tropical, low-income countries.
